# Usefulness of the Spanish version of the mood disorder questionnaire for screening bipolar disorder in routine clinical practice in outpatients with major depression

**DOI:** 10.1186/1745-0179-4-14

**Published:** 2008-05-22

**Authors:** Consuelo de Dios, Elena Ezquiaga, Aurelio García, José Manuel Montes, Caridad Avedillo, Begoña Soler

**Affiliations:** 1 Department of Psychiatry, La Paz University Hospital, Madrid, Spain; 2 Department of Psychiatry, La Princesa University Hospital, Madrid, Spain; 3 San Blas Mental Health Center, Madrid, Spain; 4 Department of Psychiatry, Sureste Hospital Madrid, Spain; 5 E-C-BIO, Madrid, Spain

## Abstract

**Background:**

According to some studies, almost 40% of depressive patients – half of them previously undetected – are diagnosed of bipolar II disorder when systematically assessed for hypomania. Thus, instruments for bipolar disorder screening are needed. The Mood Disorder Questionnaire (MDQ) is a self-reported questionnaire validated in Spanish in stable patients with a previously known diagnosis. The purpose of this study is to evaluate in the daily clinical practice the usefulness of the Spanish version of the MDQ in depressive patients.

**Methods:**

Patients (n = 87) meeting DSM-IV-TR criteria for a major depressive episode, not previously known as bipolar were included. The affective module of the Structured Clinical Interview (SCID) was used as gold standard.

**Results:**

MDQ screened 24.1% of depressive patients as bipolar, vs. 12.6% according to SCID. For a cut-off point score of 7 positive answers, sensitivity was 72.7% (95% CI = 63.3 – 82.1) and specificity 82.9% (95% CI = 74.9–90.9). Likelihood ratio of positive and negative tests were 4,252 y 0,329 respectively.

**Limitations:**

The small sample size reduced the power of the study to 62%.

**Conclusion:**

Sensitivity and specificity of the MDQ were high for screening bipolar disorder in patients with major depression, and similar to the figures obtained in stable patients. This study confirms that MDQ is a useful instrument in the daily clinical assessment of depressive patients.

## Introduction

Accurate recognition of bipolar disorders remains elusive, mainly in those patients presenting to the clinician with a major depressive episode. It has been found that almost 40% of patients suffering a major depressive episode, when carefully assessed and systematically searched for hypomania, might be more accurately diagnosed of bipolar II disorder, but only half of them were previously diagnosed by their clinicians[1].

On the other hand, use of antidepressants, mainly tricyclics or MAO inhibitors [2] and very likely dual antidepressants [3,4] has been related with manic switch, rapid cycling or worse outcome in bipolar patients. Thus, a more accurate diagnosis of those apparently "pseudounipolar" patients might lead to a more adequate treatment and subsequently contribute to reduce the personal and financial burdens related to an incorrect diagnosis [5].

A variety of instruments have been developed for the screening of hypomanic symptoms [6,7]. The Mood Disorder Questionnaire (MDQ) has been the more extensively used [8] The MDQ is a self-reported, 13-item "yes/no" questionnaire. It is easy to understand, complete and correct. The MDQ has been designed for the identification of hypomanic or manic symptoms throughout the life span. The questions are based on DSM-IV criteria and clinical experience. Furthermore, the MDQ includes a question about simultaneity of symptom occurrence and another question regarding interference with the functioning of the subject.

The MDQ has shown, in the validation carried out by his authors in a sample of psychiatric patients [8], a sensitivity of 0.73 (95% CI = 0.65–0.81) and a specificity of 0.90 (95% CI = 084–0.96) for a cut-off point score of 7 positive answers or higher. That means that seven out of 10 patients with bipolar disorder, and 9 out of 10 subjects without this disorder could be adequately classified. Several studies have been conducted with the MDQ for validation of the different language versions and with epidemiological purposes as well [9-14]. Furthermore, a version of the MDQ for the adolescent population has also been validated [15].

The validation of the Spanish version of the MDQ has been done in stable patients with a previously known diagnosis of major depression or bipolar disorder [16]. This version achieved, for a cut-off point score of 7 positive answers, similar sensitivity and specificity rates than the original version.

The purpose of this study is to evaluate the daily clinical usefulness of the Spanish version of the MDQ as a screening instrument for bipolarity in a sample of patients with a diagnosis of major depression, and not previously known as bipolar patients.

## Methods

This study has been conducted in five general psychiatry Mental Health Centers. Each investigator included new consecutive patients meeting DSM IV-TR criteria for a major depressive episode, (single o recurrent). Patients were 18 years or older. Patients with a previously known diagnosis of bipolar disorder or other psychotic disorder, depression due to a medical condition, substance abuse or dependence were excluded from the study.

Patients meeting inclusion criteria completed the Spanish version of the MDQ after signing an informed consent. Afterwards, patients were interviewed with the affective module of the SCID (First et al., 1997). Other assessment instruments used were the 17-item Hamilton Depression Rating Scale ([17,18], Young Mania Rating Scale [19] and the Global Assessment of Functioning (axis V of DSM-IV-TR).

The study protocol was approved by the Ethics Committee of the University Hospital La Paz in Madrid.

Analysis of data includes any possible association between some clinical variables and unipolar/bipolar disorder diagnosis according to SCID. Chi-square test was used for qualitative variables, while a difference in mean t-test was employed for quantitative ones.

For the MDQ, sensitivity and specificity were analyzed and plotted as a receiver-operating-characteristics curve. SCID was used as the gold standard. A Mood Disorder Questionnaire screening score of 7 or more items was used, following the recommendation of its authors [8]. Besides, the likelihood ratios for a positive and a negative test were calculated. This statistics would be preferred, instead of predictive values, because they are independent of the prevalence of the disease in the population. Larger values of a positive likelihood ratio indicate a better capacity to diagnose the illness. The smaller likelihood ratio of a negative test indicates a higher chance of being a true negative.

All the statistical analyses were performed using SPSS (version 14.0).

## Results

A total of 90 patients were included in the study. Three patients were excluded of the analysis because of a major protocol violation. According to the SCID, 87.4% (n = 76) of the sample population was classified as unipolar, whereas 12.6% (n = 11) were diagnosed of bipolar disorder.

Table 1 shows sociodemographic and clinical features of the sample population. A longer time from onset of the illness to the diagnosis in those patients suffering bipolar depression (10,9 years) was the only statistically significant difference observed (p = 0.004; 95% CI = 3.6–18.5).

**Table 1:** Sociodemographic and Clinical Characteristics of the Sample Population

		Diagnosis according to SCID	
		
Characteristics		Unipolar N = 76	Bipolar N = 11
Sex: male; N (%)		21 (27.6)	4 (36.4)
Age; Mean (SD)		45.0 (12.0)	40.0 (13.0)
Age of onset; Mean (SD)		28.2 (9.9)	23.5 (7.5)
Years of evolution from the first episode; Mean (SD)		7.4 (9.2)*	18.4 (12.9)*
History of previous episodes; N (%)		37 (48.7)	9 (81.8)
Family history of bipolar disorder; N (%)		7 (9.3)	2 (18.2)
HDRS score; Mean (SD)		23.5 (4.8)	22.2 (5.8)
YMRS score; Mean (SD)		2.3 (1.6)	2.9 (2.0)
GAF score; Mean (SD)		63.0 (13.2)	60.9 (10.7)

Diagnosis according to MDQ	Positive; N (%)	13 (61.9)	8 (38.1)
			
	Negative; N (%)	63 (95.5)	3 (4.5)

*p < 0.05.

HDRS: Hamilton Depression Rating Score; YMRS: Young Mania Rating Score;

GAF: Global Assessment Functioning; MDQ: Mood Disorder Questionnaire.

**Figure 1:**
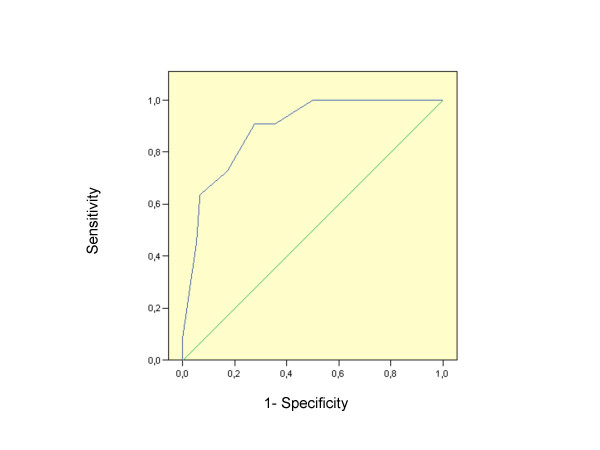
ROC curve for MDQ classification respect to SCID.

For a cut-off point score of 7 positive answers, MDQ screened 24.1% of the total sample as bipolar. Mean number of positive answers were 3.8 (SD 2.6) and 8.0 (SD 2.3) in the unipolar and bipolar subgroups of patients respectively, (*p *< 0.0001). For the cut-off point score of 7, sensitivity was 72.7% (95% CI = 63.3 – 82.1), and specificity 82.9% (95% CI = 74.9–90.9). The analysis of the Receiver Operating Characteristic (ROC) curve (Fig. 1) gives an area under the curve value of 0.778 (95% CI = 0.617–0.94). Likelihood ratio of positive test and likelihood ratio of negative test were 4.252 and 0.329 respectively.

## Discussion

In this study we have assessed the performance of the Spanish version of the MDQ for screening bipolar disorder in a sample of patients with a major depressive episode and an unknown previous diagnosis of bipolar disorder. Regarding sensitivity, our results are similar to those reported in the studies conducted for the validation of the original [8] and the Spanish [16] version of MDQ. Nevertheless, a lower specificity was obtained in our study compared with previous studies (82.9% vs. 90%).

Epidemiological data showing the prevalence of bipolar disorder in patients with a major depressive episode are controversial [1,20]. Thus, using likelihood ratios, a measure which does not depend on pre-test probability of disease, might more accurately express the usefulness of MDQ. In our study, negative likelihood ratio was low, suggesting that MDQ may be an effective instrument for detecting those depressive patients who are not very likely bipolar. This finding could have a relevant clinical application because bipolar disorder could be ruled out with fairly high degree of confidence in those patients with a major depressive episode and a negative result in the MDQ. Thus, use of antidepressants – at present of first choice in unipolar but not in bipolar depression – could be safer. On the other hand, a positive result in the MDQ requires a more detailed clinical evaluation. We agree with the author of the MDQ [21], that a clinical confirmation of bipolar disorder is always mandatory, since MDQ may wrongly identify a patient as bipolar when that is not the case. In our study the MDQ screened 24.1% of a clinical sample of patients with major depression as bipolar. This result is similar to those reported in another study conducted in Spain with patients suffering from major depression[22].

Nevertheless, SCID may miss some patients who are actually bipolar. This is mostly related to the inherent limitations of the nosological criteria used that are not considering many clinical expressions of bipolarity. In this sense, clinicians will ultimately improve the usefulness of screening instruments through their expertise in recognizing present and past manifestations of bipolarity[23]. In spite of this, the inclusion of an instrument like MDQ for screening bipolarity in the daily clinical practice might be very useful in the assessment of patients with a major depressive episode.

This study has some limitations. The estimate of the sample size was performed based on the prevalence of bipolar disorder observed by Hantouche et al. (1998)[1] in a population with major depression. Since the prevalence of bipolarity in our sample was significantly lower than expected, the power of the study was reduced to 62%. Nevertheless, sensitivity and specificity of the MDQ achieved the level reported in earlier studies.

Further studies are needed to replicate and add new data about the usefulness of the MDQ as a screening instrument for bipolar disorder in depressive patients.

## Abbreviations

MDQ: Mood Disorder Questionnaire; ROC: Receiver Operating Characteristic Curve; SCID: Structured Clinical Interview; CI: Confidence Interval; SD: Standard Deviation.

## Competing interests

Consuelo de Dios has acted as a consultant for BMS, and has been hired as a speaker or received grants by Pfizer, Astra-Zeneca, Eli Lilly, Lundbeck, Janssen Cilag, Sanofi Aventis, Juste.

Jose M. Montes has acted as a consultant for BMS, Boehringer-Ingelheim, and received honoraria from Pfizer and Astra-Zeneca.

Elena Ezquiaga has been hired as a speaker or received grants by Astra, Eli Lilly, Lundbeck, Wyeth and Juste.

Aurelio García has been hired as a speaker for Eli Lilly, Lundbeck, and received grants from Astra.

## Authors' contributions

CD conceived of the study, participated in its design and coordination, acquisition of data, analysis and interpretation of data and. drafted the manuscript, EE participated in the analysis and interpretation of data and helped to draft the manuscript, AG, JM and CA participated in the acquisition of data and helped to draft the manuscript, BS participated in the design of the study and performed the analysis and interpretation of data. All authors read and approved the final manuscript
